# Time-Series Analysis for the Number of Foot and Mouth Disease Outbreak Episodes in Cattle Farms in Thailand Using Data from 2010–2020

**DOI:** 10.3390/v14071367

**Published:** 2022-06-23

**Authors:** Veerasak Punyapornwithaya, Pradeep Mishra, Chalutwan Sansamur, Dirk Pfeiffer, Orapun Arjkumpa, Rotchana Prakotcheo, Thanis Damrongwatanapokin, Katechan Jampachaisri

**Affiliations:** 1 Center of Excellence in Veterinary Public Health, Faculty of Veterinary Medicine, Chiang Mai University, Chiang Mai 50100, Thailand; veerasak.p@cmu.ac.th; 2 Excellence Center in Veterinary Bioscience, Chiang Mai University, Chiang Mai 50100, Thailand; 3 College of Agriculture, Jawaharlal Nehru Krishi Vishwavidyalaya, Powarkheda, Narmadapuram 461110, India; pradeepjnkvv@gmail.com; 4 Akkhraratchakumari Veterinary College, Walailak University, Nakorn Si Thammarat 80160, Thailand; chakream@gmail.com; 5 Centre for One Health, Walailak University, Nakhon Si Thammarat 80161, Thailand; 6 Centre for Applied One Health Research and Policy Advice, City University of Hong Kong, Hong Kong SAR, China; pfeiffer@cityu.edu.hk; 7 Department of Pathobiology and Population Sciences, Royal Veterinary College, London, NW1 0TU, UK; 8 The 4th Regional Livestock Office, Mueang Khon Kaen, Khon Kaen 40260, Thailand; arjkumpa@hotmail.com; 9 Bureau of Disease Control and Veterinary Services, Department of Livestock Development, Bangkok 10400, Thailand; dr_sam339@hotmail.com; 10 Department of Mathematics, Faculty of Science, Naresuan University, Phitsanulok 65000, Thailand

**Keywords:** foot and mouth disease, outbreak, time-series model, forecast, trend, seasonality, endemic settings

## Abstract

Thailand is one of the countries where foot and mouth disease outbreaks have resulted in considerable economic losses. Forecasting is an important warning technique that can allow authorities to establish an FMD surveillance and control program. This study aimed to model and forecast the monthly number of FMD outbreak episodes (n-FMD episodes) in Thailand using the time-series methods, including seasonal autoregressive integrated moving average (SARIMA), error trend seasonality (ETS), neural network autoregression (NNAR), and Trigonometric Exponential smoothing state–space model with Box–Cox transformation, ARMA errors, Trend and Seasonal components (TBATS), and hybrid methods. These methods were applied to monthly n-FMD episodes (n = 1209) from January 2010 to December 2020. Results showed that the n-FMD episodes had a stable trend from 2010 to 2020, but they appeared to increase from 2014 to 2020. The outbreak episodes followed a seasonal pattern, with a predominant peak occurring from September to November annually. The single-technique methods yielded the best-fitting time-series models, including $SARIMA\left(1,0,1\right){\left(0,1,1\right)}_{12}$, $NNAR{\left(3,1,2\right)}_{12}$*,* ETS(A,N,A)*,* and TBATS(1,{0,0},0.8,{<12,5>}. Moreover, SARIMA-NNAR and NNAR-TBATS were the hybrid models that performed the best on the validation datasets. The models that incorporate seasonality and a non-linear trend performed better than others. The forecasts highlighted the rising trend of n-FMD episodes in Thailand, which shares borders with several FMD endemic countries in which cross-border trading of cattle is found common. Thus, control strategies and effective measures to prevent FMD outbreaks should be strengthened not only in Thailand but also in neighboring countries.

## 1. Introduction

The World Organization for Animal Health (OIE) lists foot and mouth disease (FMD) as an important transboundary disease [1]. The disease is caused by the FMD virus (FMDV belonging to the genus *Aphthovirus* of the *Picornaviridae* family), which affects cloven-hoof animals [2]. FMD results in economic losses due to decreased production of cattle in endemic regions and trade restrictions on disease-affected countries imposed by disease-free countries [3,4]. FMD outbreaks have been reported on several continents, including Europe, Africa, South America, and Asia [2,5,6,7,8,9,10,11]. FMD is endemic in several countries in Asia. The main serotypes of FMDV circulating in this region are O, A, and Asia 1 [12,13]. Numerous countries in Southeast Asia, including Cambodia, Lao PDR, Malaysia, Myanmar, Thailand, and Vietnam, are endemic to FMD [9,13,14]. Between 2007 and 2017, a total of 4961 FMD outbreaks were reported in these countries [15]. In Thailand, FMD has persisted for more than 60 years and spread periodically and temporarily in several regions of the country [16,17,18,19,20,21]. More than 1200 FMD outbreaks in cattle farms across several regions were officially reported from 2008 to 2019 [16]. In Southeast Asia, Thailand plays an important part in the regional FMD epidemiology due to the movements of livestock by trading with several countries, including Laos PDR, Cambodia, Vietnam, and China [22].

Time-series modeling has demonstrated its usefulness for event prediction [23,24,25]. At present, a wide range of time-series methods has been used in several epidemiological studies, resulting in predictive models that can be applied to a variety of data [25,26,27,28]. The time-series models were considered in this study for their capability to manage seasonality time-series data due to seasonal FMD outbreaks in Thailand [16]. Accordingly, (i) seasonal autoregressive integrated moving average (SARIMA) [29], (ii) error trend and seasonality (ETS) [30], and (iii) trigonometric exponential smoothing state–space model with Box–Cox transformation and an autoregressive moving average error, trend, and seasonality (TBATS) [30,31] methods were used together in the present study. Moreover, the application of neural network nonlinear autoregression (NNAR), a widely used machine learning method for nonlinear time-series data, was explored because FMD outbreak data may contain nonlinear patterns.

The SARIMA modeling approach is widely used in various disciplines and is regarded as one of the most efficient methods of modeling time-series data with a seasonal trend [29]. SARIMA has the advantage of requiring few model parameters for modeling and prediction. Similar to SARIMA, the ETS can deal with seasonality data. When dealing with data that exhibits heterogeneity and non-linearity, the ETS offers an advantage over SARIMA. The artificial neural network is a machine learning algorithm based on the idea of a neuron from a human brain [32,33,34]. As a result, the computer machine is able to learn and make decisions in a human-like fashion [35]. Nonlinear autoregression (NNAR) is a type of artificial neural network that uses lagged values of a time series as input to a neural network. This method incorporates the most recent observations from the same season when used with seasonal data [29]. The main difference between NNAR and SARIMA models is that NNAR can manage nonlinear sequences of time-series data better than ARIMA [33], whereas SARIMA has a better capability to take account of a linear pattern in data than NNAR. Furthermore, it is possible to model time series with different seasonality using TBATS [30,31]. With this method, a Fourier series-based trigonometric representation is used to model seasonality. The advantage of this method is the capacity to model seasonal effects of non-integer lengths [36]. Aside from simple time-series models, hybrid models that integrate forecasting methods are increasingly being used in a variety of disciplines because they typically outperform single methods [28,34,37]. In the current study, the predictive abilities of hybrid models based on the combination of SARIMA, NNAR, ETS, and TBATS, such as SARIMA-NNAR, ETS-TBATS, and NNAR-TBATS, were assessed. Supplementary Materials Table S1 provides a brief overview of the models used in this study, including their applications, assumptions, and limitations.

In epidemiology, the development of a reliable method for the explanation and prediction of FMD outbreaks is of utmost importance. However, only a few studies have used time-series methods to explore and forecast FMD outbreaks [7,38,39,40]. A lack of outbreak prediction may pose some challenges in the formulation of an effective FMD surveillance and control program in Thailand. Furthermore, a better understanding of FMD outbreaks in one country can support other countries in developing an effective control program to prevent FMD from spreading across borders. Thus, given Thailand’s proximity to other FMD-endemic countries, the findings of this study, which are based on 10 years of retrospective data, will be useful to livestock authorities and stakeholders in those boundary countries.

The objectives of this study were to examine FMD epidemic episodes (n-FMD episodes) in Thailand over a 10-year period for the presence of trend and seasonality using time-series methods and to compare the prediction performances of time-series forecasting models for prospective estimation of FMD outbreak occurrences.

## 2. Materials and Methods

### 2.1. Outbreak Episode Definition, Data, and Data Analysis Steps

An outbreak episode of FMD was defined as an official report of an FMD outbreak in an outbreak area where disease investigation and FMD disease confirmation were undertaken by an authority from the Department of Livestock and Development (DLD) [19]. Notably, the episode of FMD outbreak defined as the unit of analysis in this study is similar to the unit utilized in several previous studies [7,9,41].

Data of n-FMD episodes in Thailand from 2010 to 2020 (n = 1209 episodes) were obtained from the DLD. Time-series data were generated from the original data. Further, the data was divided into two parts: data from 2010 to 2019 (Data: 2010–2019; training data) and data from 2020 (Data: 2020; validation data), with the former being used for model development and the latter for model validation, respectively.

**Figure 1:**
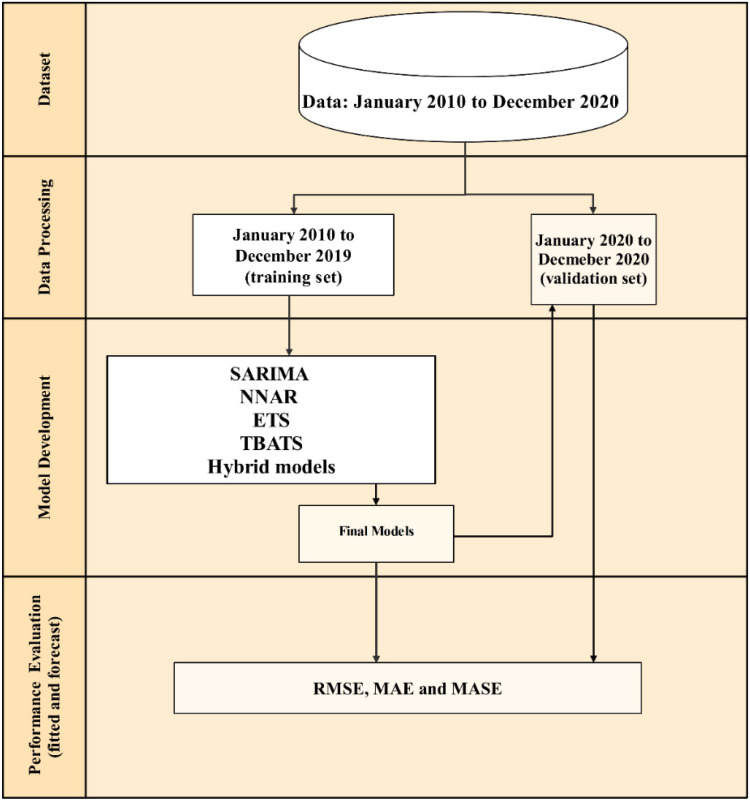
Time-series modeling procedure. A full dataset of the number of FMD outbreak episodes was split into training and validation datasets. Forecast models were developed using seasonal autoregressive integrated moving average (SARIMA), error trend seasonality (ETS), neural network autoregression (NNAR), and Trigonometric Exponential smoothing state–space model with Box–Cox transformation, ARMA errors, Trend and Seasonal components (TBATS), and hybrid methods. With the validation data, error measures, including root mean squared error (RMSE), mean absolute error (MAE), and mean absolute scaled error (MASE), were determined in order to compare the performances of prediction models.

Data analysis and time-series modeling were performed using R statistical software [42] and “xts”, “tsbox”, “plotly”, “TSstudio”, “forecast”, and “forecast Hybrid” packages. The step of time-series data analysis and modeling includes (i) decomposition of time-series data into several components, (ii) determination of the final model through the model development process, (iii) evaluation of the performances of the final model developed, and (iv) forecasting the n-FMD episodes from the final model. These steps are depicted in Figure 1.

### 2.2. Decomposition of Time-Series Data

An additive decomposition of the FMD time series was carried out to describe the trends and seasonality components using R statistical software version 4.0.4 [42]. At this step, time-series data were decomposed into trend, seasonal, and residual components. The model is given as follows:

(1) $$Y_{t}=T_{t}+S_{t}+I_{t}\;$$

where $Y_{t}$ is the n-FMD episode at time t, $T_{t}$ is the trend-cycle component at time t, $S_{t}$ is the seasonal component at time t, and $I_{t}$ is the irregular component at time t.

### 2.3. Model Development

#### 2.3.1. SARIMA Model

The SARIMA is composed of seasonal and non-seasonal components. The general SARIMA model [29] has the following form:

(2) $$\varphi \left(X\right)\Phi \left(X^{12}\right)\Delta^{d}\Delta_{12}^{D}Z_{t}=\theta \left(X\right)\Theta \left(X\right)a_{t}$$

where φ(X) = non-seasonal autoregressive operator of order p, θ(X) = non-seasonal moving average operator of order q, $\Phi \left(X^{12}\right)\;$= the autoregressive seasonal operator of order P, Θ(X) = the moving averages operator of order Q, $\Delta^{d}\;$= the operator difference, $\Delta_{12}^{D}\;$= operator seasonal difference, and $a_{t}\;$= white noise.

The expression of SARIMA is given as

(3) $$ARIMA\left(p,d,q\right){\left(P,D,Q\right)}_{s}$$

where p = autoregressive order in non-seasonality, d = difference in non-seasonality, q = the non-seasonal moving average order, P = autoregressive order in seasonality, D = differences in seasonality, Q = moving average order in seasonality, and s = length of the seasonal pattern (s = 12 in this study).

The development of SARIMA models was carried out using auto.arima( ) function from a “forecast” package. This function determined the p,d,q, P,D,Q for several candidate models. Akaike information criterion (AIC) was used as criteria to verify the model that best fit the data.

The *auto.arima* function utilized the Hyndman–Khandarkar algorithm for automatic autoregressive integrated moving average (ARMA) modeling. As previously described, this algorithm performed various steps in the model selection procedure [29,43]. Based on such procedures, the final model was defined as the model with the lowest AIC. Additionally, the model assumptions were checked by examining the standardized residual plot, autocorrelation function plot of residuals, and the *p*-values plot from the Ljung–Box test.

#### 2.3.2. NNAR Model

The NNAR used lagged values of the time series as input to a neural network. A network of three layers of functioning is linked by acyclic linkages. The equation of the NNAR is as follows [44]:

(4) $$y_{t}=\omega_{0}+\overset{Q}{\underset{j=1}{\sum }}\omega_{g}g\left(\omega_{oj}+\overset{p}{\underset{i=1}{\sum }}\omega_{i,j}y_{t-1}\right)+e_{t}$$

where *y_t_* and $\left(y_{t-i,...,}y_{t-p}\right)$ are the output and the input, $\omega_{i,j}\left(i=0,1,2,...,P,\;j=1,2,...,Q\right)$ and $\omega_{j}\left(j=0,1,2,...,Q\right)$ are model parameters, which are known as connection weights; the number of input nodes is represented by P while the number of hidden nodes is indicated by Q.

For seasonal data, the NNAR model [29] can be written as

(5) $$\begin{array}{c} NNAR{\left(p,P,K\right)}_{m} \end{array}$$

where p = the last observed values from the same season using as inputs, P = lagged inputs, K = number of neurons (nodes) in the hidden layer, and m = the number of months.

A function *nnetar* from “forecast” package was used to fit $NNAR{\left(p,P,K\right)}_{m}$ model. For seasonal time series, P = 1 was set as default value and p was chosen from the optimal linear model fitted to the seasonally adjusted data. We used the default setting, and thus K=(p+P+1)/2 was set using the nnetar( ) function [29].

#### 2.3.3. ETS Model

Regarding a state–space framework, the ETS combines error (E), trend (T), and seasonal (S) components in a smoothing calculation. The ETS offers a total of 30 possible ETS combinations; thus, it has the ability to analyze a variety type of time-series data, even with both heterogeneity and non-linearity. Within a state–space framework, the E component is either additive (A) or multiplicative (M), T and S components may be A, M, or inexistent (N), and T is referred to as dampened additively ($A_{d}$) or multiplicatively $\left(M_{d}\right)\;$ [45]. The state–space equations can be written as [46]

(6) $$y_{t}=w\left(x_{t-1}\right)+r\left(x_{t-1}\right)\varepsilon_{t}$$

(7) $$x_{t}=f\left(x_{t-1}\right)+g\left(x_{t-1}\right)\varepsilon_{t}$$

where w, f, and g are coefficient while $\varepsilon_{t}$ represents the Gaussian white noise series. Equation (6) is known as the observation that describes the relationship between the observation $x_{t-1}$ and $y_{t}$. Equation (7) is the transitional equation describing the evolution of states over time. The ETS model was performed using the ets( )  function from the “forecast” package.

#### 2.3.4. TBATS Model

Multiple seasonal incorrect cycles can be accommodated by TBATS. A combination of Fourier with an exponential smoothing state–space model and a Box–Cox transformation is represented by the TBATS models in this study. The basic equation of the TBATS took the following form [30,47]:

(8) $$y_{t}^{\left(\omega \right)}=l_{t-1}+\phi b_{t-1}+\overset{T}{\underset{t=1}{\sum }}s_{t-m_{i}}^{\left(i\right)}+d_{t}$$

where $y_{t}^{\left(\omega \right)}$ indicates the Box–Cox transformation parameter (ω) and $y_{t}$ is the observation at time t, $l_{t}$ is the local level, ϕ denotes the damped trend, b denotes the long-run trend, T indicates the seasonal pattern, $s_{t_{i}}^{\left(i\right)}$ denotes the ith seasonal component, $m_{i}$ indicates the seasonal periods, and $d_{t}$ is an ARMA(p,q) process for residuals. The TBATS model was identified by tbats( ) function included in the “forecast” package.

#### 2.3.5. Hybrid Model

Time-series hybrid models including SARIMA-NNAR, SARIMA-ETS, SARIMA-TBATS, NNAR-ETS, NNAR-TBATS and ETS-TBATS were developed using functions from the “forecast Hybrid” package and R. The functions offer the automated procedure to obtain the best fitting model. The detail of model development from the functions was previously described [8,48].

### 2.4. Forecast and Model Performances

Time-series models developed from Data: 2010–2019 were used to predict n-FMD episodes in 2020. By comparing forecast and actual n-FMD episode values, the forecasting ability was evaluated.

We used several evaluation error metrics, including mean absolute error (MAE), root mean squared error (RMSE), and mean absolute scaled error (MASE) [29,49], to determine prediction performances among models developed from the full and training datasets [29]. It was important to note that a mean absolute percentage error (MAPE), which was generally reported, was unable to be determined as our data contained zero counts for some months of time-series data. Therefore, the scaled errors such as MASE were proposed as an alternative to using MAPE. Similar to MAPE, the MASE is independent of the scale of the data [29]. The MASE is considered the most versatile and reliable measure of forecast accuracy and can be used to compare forecast accuracy both on single and multiple time series [30].

The MAE and RMSE are expressed as follows:

(9) $$MAE=\frac{1}{n}\overset{n}{\underset{t=1}{\sum }}|Y_{t}-{\hat{Y}}_{t}|$$

(10) $$RMSE=\sqrt{\frac{1}{n}\overset{n}{\underset{t=1}{\sum }}|Y_{t}-{\hat{Y}}_{t}{|}^{2}}$$

(11) $$MASE=\frac{1}{n}\sum_{t=1}^{n}\left(\left|y_{t}-{\hat{y}}_{t}\right|\;/\;\frac{1}{n-1}\sum_{t=2}^{n}\left|y_{t}-{\hat{y}}_{t}-1\right|\right)$$

where $y_{t}$ denotes the actual values, ${\hat{y}}_{t}$ represent the predicted values, and n indicates the number of observations.

It is generally accepted that the lower the error metrics, the better the method [30,49]. All error metric values were calculated for models developed from Data: 2010–2019 to assess the accuracy of such models for in-sample data (training data), whereas these values were also determined to assess how the models developed from the training data performed on out-of-sample data (validation data; Data: 2020).

In addition, we also forecasted the n-FMD episodes for the period of 2021–2023 based on 2010–2020 FMD data.

## 3. Results

### 3.1. Trends and Seasonality

**Figure 2:**
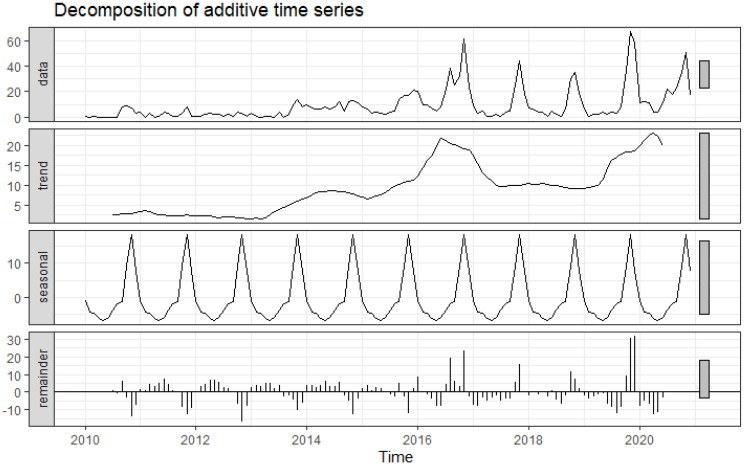
Decomposition of the number of time-series FMD outbreak episodes from January 2010 to December 2020 in actual (data), trend, decomposed seasonal trait (seasonal), and random fluctuation (remainder) of FMD outbreak episodes were illustrated.

From 2013 to 2019, there was a seasonal increase in the number of n-FMD episodes. The year with the most episodes was 2016 (Figure 2). There was a downward trend in n-FMD episodes from mid-2016 to mid-2017. From late 2018 to mid-2019, the n-FMD episodes appeared to be stable. Following that, there was an upward trend in the number of n-FMD episodes.

The seasonal pattern demonstrated that the peak of n-FMD episodes was mainly observed from September to December annually.

### 3.2. Fitted Time-Series Models, Model Performances, and Forecasts

**Figure 3:**
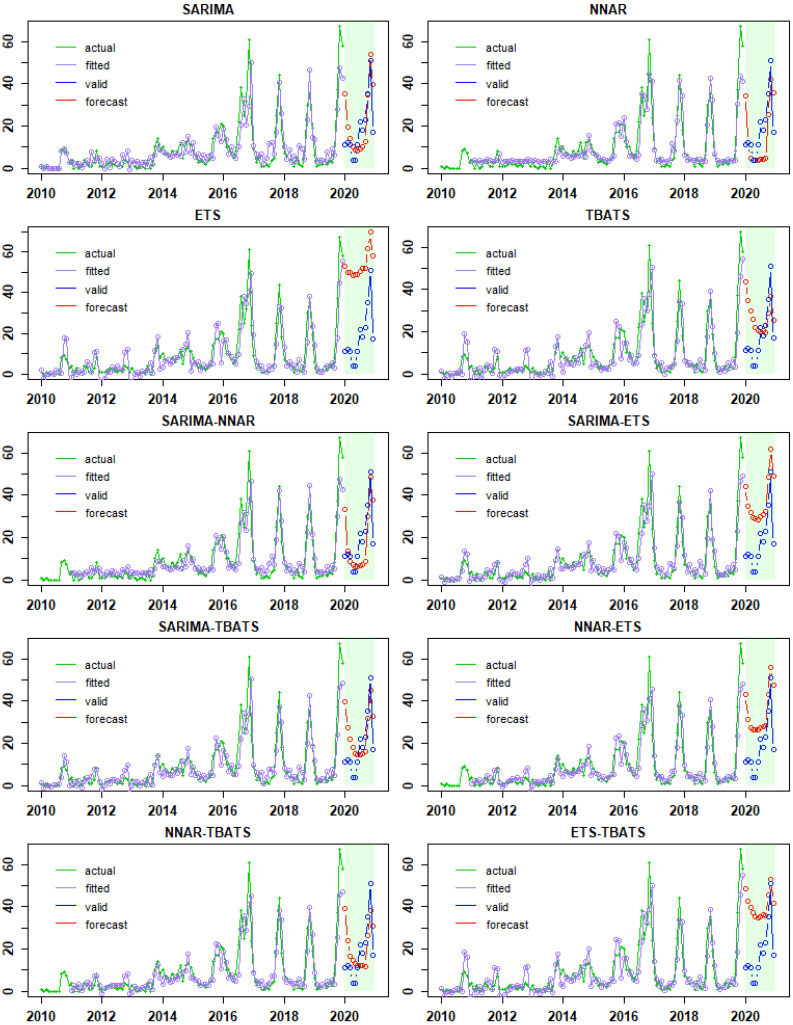
Actual, fitted, and forecast value from SARIMA, NNAR, ETS, TBATS. SARIMA-NNAR, SARIMA-ETS, SARIMA-TBATS, NNAR-ETS, NNAR-TBATS, and ETS-TBATS models. The x-axis and y-axis are the year and number of FMD outbreak episodes, respectively.

For the entire dataset, the program generated and tested 192 potential models, $SARIMA\left(1,0,1\right){\left(0,1,1\right)}_{12}$ was the best-fitting model with the lowest AIC. Assumptions for model residuals were tested before this model could be used to forecast (Figure 3). The ACF of residuals showed no significant deviation from a zero-mean white noise process, and all *p* values for the Ljung–Box statistic were large (*p* > 0.05), indicating no violations. The best forecasting model for NNAR was found to be $NNAR{\left(3,1,2\right)}_{12}$, whereas the fitted model for ETS was ETS(A,N,A). The fitted model for TBATS was TBATS(1,{0,0},0.8,{<12,5>}).

**Figure 4:**
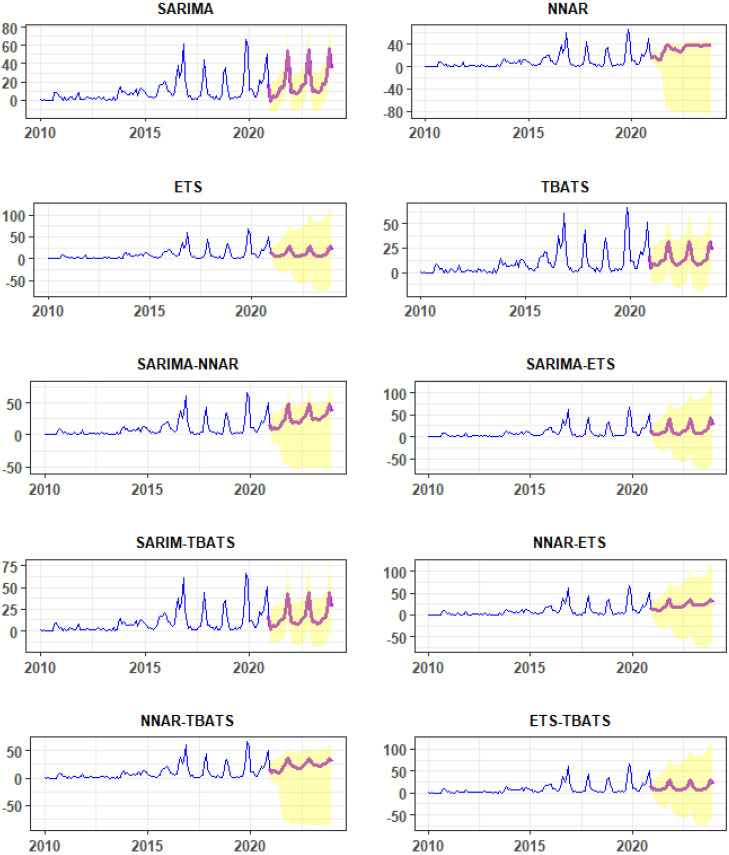
Forecasts of the number of FMD episodes (red line) from SARIMA, NNAR, ETS, TBATS, SARIMA-NNAR, SARIMA-ETS, SARIMA-TBATS, NNAR-ETS, NNAR-TBATS, and ETS-TBATS models. The x-axis and y-axis are the year and number of FMD outbreak episodes, respectively. The yellow band indicates a 95% confidence interval of forecast values.

Figure 3 shows the actual and fitted values for each final model derived from the training data (Data: 2010–2019). Furthermore, actual and forecast values from the final models tested with validation data (Data: 2020) were illustrated (Figure 4). The projection appearance of actual and fitted values among models appears to be similar graphically (Figure 3), whereas actual and forecast values of the n-FMD episodes appear to differ among models (Figure 4). Additionally, the forecast values of n-FMD episode from each model are presented in Supplementary Materials Table S2.

In terms of RMSE, MAE, and MASE (Table 1), it was found that using NNAR for training data produced results that outperformed those obtained from other single-technique methods. On the other hand, the lowest values of the RMSE, MAE, and MASE from testing data demonstrate that SARIMA is more accurate than other competing models in terms of accuracy. In hybrid models, the SARIMA-NNAR and NNAR-TBATS models outperformed the others.

## 4. Discussion

In the present study, we applied the time-series method to FMD outbreak data to determine epidemiological features and explore the predictivity in forecasting the n-FMD episodes through various time-series models. Based on the most recent outbreak data used in this study, forecasts of n-FMD episodes for the following three years were also projected.

From 2014 to 2019, there was an increasing trend of n-FMD episodes in Thailand, which was in agreement with a previous report [16]. The increasing trend could be attributed to the presence of more than one serotype of FMD virus causing outbreaks. In general, FMD virus serotype O was responsible for the majority of the FMD outbreaks in Thailand each year [16,17]. However, FMD virus serotype A has become more prevalent since 2013. During the years 2013–2015, both serotypes A and O were responsible for a number of FMD outbreaks in different parts of the country [16]. It has been discussed that the presence of two serotypes causing outbreaks nationwide might pose a difficulty in preventing and controlling the disease [17,50]. The variation in the genetics of FMD virus serotype A is a major concern because the ratio of antibody titer against the heterologous field strain and antibody titer against the homologous vaccine strain or r-value [51] is not high, indicating that a moderate-level matching is observed between FMD vaccine strains and FMD outbreak strains, especially in 2016, 2017, and 2019 [17]. Given that, it is likely that vaccines used in some areas could not adequately provide disease protection [17]. Another explanation for the increase in n-FMD episodes is the enhancement of surveillance programs by DLD, which encourages farmers to report FMD outbreaks to DLD officers [50].

Our findings revealed a seasonal variation, indicating that the FMD outbreak in Thailand occurred on a regular basis. A predominant peak of reported FMD outbreaks was observed between October to December annually. This finding could be due to the possibility that climatic influences cause stress in cattle as they transition from the rainy season to the winter season, and thus, they are more susceptible to the disease [50]. Moreover, the high degree of trade activity throughout the last 3 months of the year might potentially have a role in the spreading of the disease across locations [50]. The finding of the seasonality of n-FMD episodes in this study was in agreement with several studies conducted in Thailand [52,53], Vietnam [9], and Colombia [41] but different from a report in Ethiopia that showed no seasonal trend for FMD outbreaks [7].

There is a lot of ambiguity and nonlinearity in FMD epidemic data. Unsatisfactory results may be obtained by predictive models that fail to account for seasonal fluctuation and nonlinearity [54]. Thus, SARIMA, ETS, and TBATS methods that account for seasonal variation, the nonlinear NNAR approach that incorporates seasonality and nonlinearity in its algorithm, and hybrid models that combine some of the advantages of these methods were utilized. According to our findings, NNAR outperformed other single-technique methods. Better performance of NNAR may be due to its ability to take account of the nonlinear characteristics of n-FMD episodes exhibited during some parts of the study period. However, with validation data, the SARIMA model outperformed other single-technique methods in terms of validity. This could be because SARIMA appears to be an effective model for short-term forecasting [26,27]. However, a model’s performance changes depending on the data it utilizes. Thus, we propose examining both SARIMA and NNAR predictions together for predicting future n-FMD episodes because they each have their own set of advantages. Furthermore, our findings demonstrated that the prediction ability from the single-technique methods and hybrid methods was likely to be similar. Nevertheless, it should not be extrapolated that hybrid models will not improve prediction ability for other FMD outbreak data or other data types because several studies from various fields have shown that hybrid models outperform non-hybrid models [28,33,44,55]. As addressed above, the performance of time-series methods is varied depending on characteristics and nature of the data. Thus, we suggest building multiple models from the same dataset to get better predictive insights.

Furthermore, it is generally recommended that the time-series method should be used on an updated dataset [40]. Hence, we recommend developing a time-series model to forecast n-FMD episodes on a regular basis, such as every six months or every year, for continuous projections and better prediction.

This study has some limitations to be considered. First, the FMD dataset is likely to be subjected to underreporting bias, as also indicated by other studies conducted in Thailand [56] and elsewhere [57]. Second, we did not determine temporary patterns for n-FMD episodes at provincial or regional levels due to the fact that the majority of the monthly FMD episodes data would contain zero values, which may not be appropriate for SARIMA and NNAR. For follow-up studies, other time-series methods should be investigated. In addition, FMD outbreak data from several countries in the region should be analyzed.

There is evidence that the same FMDV strains (e.g., O/EA/Mya-98, O/Middle East-South Asia, and O/ME-SA/Ind-2001) circulate in different Southeast Asian countries [8], suggesting that regional cooperation is required to control disease spread across borders. The lack of effective outbreak alerts and FMD outbreak data sharing among Southeast Asian endemic countries is one of the most difficult challenges for FMD prevention [58]. The analysis of retrospective time-series data and the prediction of future FMD outbreak occurrences in Thailand in this study would provide important information and warning messages to FMD endemic neighboring countries. The prediction of future n-FMD episodes would support the existing livestock surveillance system [16]. Livestock authorities can use the prediction from time-series models to develop an additional strategic plan for relevant control strategies for future FMD outbreaks, such as an implementation of rigorous animal movement control during the last three months of the year. Furthermore, in the Southeast Asia region, strict quarantine procedures should be implemented when cattle are transported across international borders. We also recommended greater regional cooperation in enhancing FMD prevention and controls.

## 5. Conclusions

To our knowledge, this is the first study to use the SARIMA, ETS, NNAR, TBATS, and hybrid models for the n-FMD episodes in Thailand to describe trends and seasonality as well as forecast the future n-FMD episodes. This study provides a better understanding of FMD outbreaks in Thailand in terms of trend, seasonality, and forecast based on 10-year outbreak data. The approaches demonstrated in this study could be used by livestock authorities to forecast FMD outbreak episodes in the short and long term and thus design or improve the FMD control strategies to prevent future outbreaks. The results of the study were not limited to Thailand; neighboring countries could use our findings and predictions as basic information to develop a regional strategy to mitigate FMD outbreaks in the region.

## Figures and Tables

**Table 1:** Error matrices for time-series models applied to training and validation datasets.

Model ^1^	Training Data (Data: 2010–2019)			Validation Data (Data: 2020)		
	RMSE ^2^	MAE	MASE	RMSE	MAE	MASE
SARIMA	6.21	3.81	0.64	11.47	8.75	1.47
NNAR	5.21	3.39	0.56	12.61	10.46	1.76
ETS	6.59	4.44	0.75	36.11	35.26	5.94
TBATS	6.38	4.26	0.89	16.24	13.38	2.25
SARIMA-NNAR	5.59	3.63	0.61	11.43	8.79	1.48
SARIMA-ETS	5.94	3.82	0.64	20.89	19.2	3.23
SARIMA-TBATS	5.91	3.75	0.63	12.56	10.4	1.75
NNAR-ETS	5.38	3.52	0.60	17.49	15.17	2.60
NNAR-TBATS	5.40	3.53	0.60	11.41	10.02	1.69
ETS-TBATS	6.42	4.29	0.72	24.44	22.14	3.73

^1^ SARIMA = seasonal autoregressive integrated moving average, ETS = error trend seasonality, NNAR = neural network autoregression, TBATS = Trigonometric Exponential smoothing state-space model with Box–Cox transformation, autoregressive integrated moving errors, Trend and Seasonal components. ^2^ RMSE = root mean squared error, MAE = mean absolute error and MASE = mean absolute scaled error.

## Data Availability

The data that support the findings of this study are available from the Department of Livestock Development (DLD) by restriction apply to the availability of these data, which were used under license for the current study, and so are not publicly available. Requests to access the datasets should be directed to info@dld.go.th.

## Supplementary Materials

The following supporting information can be downloaded at: https://www.mdpi.com/article/10.3390/v14071367/s1, Table S1: Comparison of Application, assumptions, and limitations of forecasting models and Table S2: Forecast values of FMD outbreak episodes during January 2021 to December 2023 [30,59,60,61,62,63,64,65,66].
