# Supplementary material for: Time-Series Analysis for the Number of Foot and Mouth Disease Outbreak Episodes in Cattle Farms in Thailand Using Data from 2010–2020
Source: Viruses. 2022 Jun 23;14(7):1367. doi: 10.3390/v14071367 (PMC9320723; doi:10.3390/v14071367)
Supplement: Supplementary file 1 [file viruses-14-01367-s001.zip › viruses-1680568-supplementary.pdf]

Table S1: Comparison of Application, assumptions, and limitations of forecasting models

Methods	Application and assumptions	limitations
**SARIMA**	-Seasonal ARIMA models can be applied with other seasonal parameters in ARIMA models through a multiplicative process commonly known as SARIMA models" (Rahman et al,.2022, 144).<br>-Modeling linear relationships in time series with singular seasonality.<br>- Stationarity is an important condition when building SARIMA models, and differentiation is often used to balance time series data". (Mishra et al,.2020, 3)	-Insufficient modeling of nonlinear relationships.<br>-The model can only handle a single seasonal effect.<br>-The length of the season should not be too long.<br>-This model requires high volume of manual work.
**ETS**	-The model contains three main components of time series: Trend (T), Seasonal (S), Error (E). (Raghav et al,.2022, 3).<br>-The model can take into account different combinations of trend and seasonal components (Perone, 2021).<br>-This model can capture the complex non-linear nature of the data series." (Mishra et al,.2023,9).<br>- Stationarity is not required, it is often easier to deal with missing data.<br>-The model outperforms other models in terms of small sample size, non-normal data distribution, non-stationarity, trend, and seasonality.	-The model does not allow adding exogenous variables to the model to improve predictions.<br>-This model cannot handle multiple seasonal patterns. (Schreiber et al,.2020, 5).
**NNAR**	- The model can handle the complex nonlinear nature of the data series. (Al Khatib et al,.2021, 272).<br>- The model can be viewed as a network of neurons or nodes representing complex nonlinear relationships and functional forms (Perone, 2021).<br>-These models differ from ARIMA models by the presence of a hidden layer, in which the linear weighted inputs are modified by nonlinear functions (Yonar et al,.2022, 107).	- The resulting model corresponds to a simple linear regression and becomes nonlinear only when an intermediate layer with "hidden neurons" is included. for seasonal data (Perone, 2021).<br>-This model requires a lot of data.<br>-The model does not allow adding exogenous variables to the model to improve predictions.
**TBATS**	- TBATS can be used to forecast time series with complex seasonal patterns. (For example, non-integer seasonality, non-nested seasonality, and large-period seasonality), with no seasonal restrictions, can generate detailed long-term forecasts.<br>-This model is preferable when seasonality changes over time.	-The model does not allow adding exogenous variables to the model to improve predictions.<br>-The method is very broad. Many candidate models are being built and evaluated behind the scenes. This results in slow computation. This can be critical when the model

	-seasonality is allowed to change slowly over time in a TBATS model. (Abotaleb, et al,.2021, 1614).<br>-The model can handle any type of autocorrelation in the residuals.<br>-The TBATS model uses trigonometric seasonal expression to separate complex seasonal components.<br>- Ability to make detailed forecasts of time series over longer periods of time.	needs to be trained on many parallel time series.<br>- This model requires huge amounts of data.
**Hybrid methods**	-Hybrid methods can improve prediction accuracy better than single original method.<br>-The hybrid methods combine forecasts from different techniques, such as ARIMA models, ETS (error, trend, seasonality) and ANN..).<br>- The combination process is based on the weight of each technique on the final predicted output; combining the best individual models with the same weights has been shown to be more appropriate than weights based on error values (Perone, 2021).	-If the time series can be assumed to be largely linear, then classical statistical methods are better than hybrid models.<br>-These models depend on the quality of combination of different models.<br>- This model requires huge amounts of data.

References:

1) Al Khatib, A. M. G., Yonar, H., Abotaleb, M., Mishra, P., Yonar, A., Karakaya, K., ... & Dhaka, V. (2021). Modeling and forecasting of egg production in India using time series models, 37, 4, 265-273 DOI: 10.15312/EurasianJVetSci.2021.352.
2) Mishra, P., Al Khatib, A. M. G., Sardar, I., Mohammed, J., Ray, M., Manish, K., ... & Rono, K. (2020). Modelling and forecasting of COVID-19 in India. Journal of Infectious Diseases and Epidemiology, 6(5), 1-11. DOI: 10.23937/2474-3658/1510162.
3) Mishra, P., Alakkari, K. M., Lama, A., Ray, S., Singh, M., Shoko, C., ... & Karakaya, K. (2023). Modeling and Forecasting of Sugarcane Production in South Asian Countries. CURRENT APPLIED SCIENCE AND TECHNOLOGY, Vol. 23 No. 1, 1-15. DOI: 10.55003/cast.2022.01.23.002.

4) Perone, G. (2021). Comparison of ARIMA, ETS, NNAR, TBATS and hybrid models to forecast the second wave of COVID-19 hospitalizations in Italy. The European Journal of Health Economics, 1-24. DOI: 10.1007/s10198-021-01347-4

5) Raghav, Y. S., Mishra, P., Alakkari, K. M., Singh, M., Al Khatib, A. M. G., & Balloo, R. (2022). Modelling and forecasting of pulses production in south asian countries and its role in nutritional security. Legume Research-An International Journal, 45(4), 454-461. DOI: 10.18805/LRF-645.
6) Rahman, U.H., Ray, S., Al Khatib, A.M.G., Lal, P., Mishra, P., Fatih, C., Williams, A.J., Karakaya, K., Shrivastri, S., and Alakkari, K. (2022). State of Art of SARIMA Model in

Second Wave on COVID-19 in India. International Journal of Agricultural and Statistical Sciences. DocID: https://connectjournals.com/03899.2022.18.141.
7) Schreiber, M. A., & Cavanaugh, C. H. (2020). The Application of Machine Learning Methods to Time Series Forecasting.
8) Yonar, H., Yonar, A., Mishra, P., Abotaleb, M., Al Khatib, A. M. G., Makarovskikh, T., & Cam, M. (2022). Modeling and forecasting of milk production in different breeds in Turkey. The Indian Journal of Animal Sciences, 92 (1): 105–111.
9) Abotaleb, M., Ray, S., Mishra, P., Karakaya, K., Shoko, C., Al khatib, A. M. G., & Balloo, R. (2021). Modelling and forecasting of rice production in south Asian countries. AMA, Agricultural Mechanization in Asia, Africa and Latin America, 51(03), 1611–1627.

Table S2. Forecast values of number of FMD episode during January 2021 to December 2023

Method	Year	Month	Point Forecast	95% lower bond	95% upper bond
SARIMA	2021	Jan	-0.92	-15.04	13.19
SARIMA	2021	Feb	2.38	-14.15	18.92
SARIMA	2021	Mar	5.20	-12.15	22.54
SARIMA	2021	Apr	3.67	-13.97	21.30
SARIMA	2021	May	4.85	-12.89	22.59
SARIMA	2021	Jun	8.86	-8.92	26.64
SARIMA	2021	Jul	14.84	-2.96	32.63
SARIMA	2021	Aug	13.95	-3.86	31.75
SARIMA	2021	Sep	17.89	0.08	35.69
SARIMA	2021	Oct	35.82	18.01	53.62
SARIMA	2021	Nov	53.76	35.96	71.57
SARIMA	2021	Dec	30.82	13.01	48.62
SARIMA	2022	Jan	8.07	-10.80	26.93
SARIMA	2022	Feb	8.43	-10.81	27.67
SARIMA	2022	Mar	9.45	-9.92	28.83
SARIMA	2022	Apr	6.83	-12.60	26.26
SARIMA	2022	May	7.35	-12.10	26.80
SARIMA	2022	Jun	10.96	-8.50	30.41
SARIMA	2022	Jul	16.68	-2.77	36.14
SARIMA	2022	Aug	15.64	-3.82	35.10
SARIMA	2022	Sep	19.49	0.03	38.95
SARIMA	2022	Oct	37.37	17.91	56.83
SARIMA	2022	Nov	55.28	35.82	74.74
SARIMA	2022	Dec	32.31	12.86	51.77
SARIMA	2023	Jan	9.55	-10.88	29.98
SARIMA	2023	Feb	9.91	-10.86	30.68
SARIMA	2023	Mar	10.93	-9.97	31.82
SARIMA	2023	Apr	8.30	-12.65	29.25
SARIMA	2023	May	8.81	-12.15	29.78
SARIMA	2023	Jun	12.42	-8.55	33.39
SARIMA	2023	Jul	18.15	-2.83	39.12
SARIMA	2023	Aug	17.11	-3.87	38.08
SARIMA	2023	Sep	20.96	-0.02	41.93
SARIMA	2023	Oct	38.83	17.86	59.81

SARIMA	2023	Nov	56.74	35.77	77.72
SARIMA	2023	Dec	33.78	12.80	54.75
NNAR	2021	Jan	17.30	2.84	32.36
NNAR	2021	Feb	18.29	-2.48	35.88
NNAR	2021	Mar	18.59	-9.23	36.61
NNAR	2021	Apr	15.31	-15.06	32.50
NNAR	2021	May	13.05	-37.27	31.46
NNAR	2021	Jun	13.21	-73.73	34.49
NNAR	2021	Jul	22.95	-82.38	42.98
NNAR	2021	Aug	26.66	-86.48	43.16
NNAR	2021	Sep	33.60	-87.84	46.88
NNAR	2021	Oct	38.19	-87.93	50.47
NNAR	2021	Nov	39.23	-77.98	51.17
NNAR	2021	Dec	31.49	-89.77	45.12
NNAR	2022	Jan	30.59	-90.78	45.51
NNAR	2022	Feb	31.17	-90.80	46.76
NNAR	2022	Mar	31.65	-90.36	47.91
NNAR	2022	Apr	28.97	-89.71	46.31
NNAR	2022	May	26.60	-91.10	45.59
NNAR	2022	Jun	25.81	-91.73	43.87
NNAR	2022	Jul	33.23	-90.68	48.39
NNAR	2022	Aug	36.90	-89.83	47.25
NNAR	2022	Sep	38.22	-89.34	49.77
NNAR	2022	Oct	38.55	-88.92	50.27
NNAR	2022	Nov	38.60	-90.60	51.19
NNAR	2022	Dec	37.89	-90.97	49.51
NNAR	2023	Jan	37.84	-92.35	50.91
NNAR	2023	Feb	37.92	-91.64	50.29
NNAR	2023	Mar	37.98	-92.17	49.28
NNAR	2023	Apr	37.59	-92.87	51.35
NNAR	2023	May	37.14	-93.11	49.49
NNAR	2023	Jun	36.96	-90.97	48.87
NNAR	2023	Jul	38.19	-91.43	50.25
NNAR	2023	Aug	38.46	-91.72	49.69
NNAR	2023	Sep	38.54	-91.02	51.78
NNAR	2023	Oct	38.56	-91.80	51.51
NNAR	2023	Nov	38.56	-91.70	51.46
NNAR	2023	Dec	38.51	-91.85	50.00
ETS	2021	Jan	10.39	-5.35	26.13
ETS	2021	Feb	7.91	-13.71	29.52
ETS	2021	Mar	7.17	-19.04	33.37
ETS	2021	Apr	5.49	-24.61	35.60
ETS	2021	May	5.32	-28.24	38.87
ETS	2021	Jun	5.57	-31.11	42.25
ETS	2021	Jul	7.90	-31.66	47.46
ETS	2021	Aug	9.41	-32.83	51.65
ETS	2021	Sep	10.44	-34.32	55.21
ETS	2021	Oct	20.84	-26.32	67.99
ETS	2021	Nov	29.84	-19.59	79.26

ETS	2021	Dec	18.29	-33.31	69.89
ETS	2022	Jan	10.39	-43.30	64.08
ETS	2022	Feb	7.91	-47.79	63.60
ETS	2022	Mar	7.17	-50.47	64.80
ETS	2022	Apr	5.49	-54.01	65.00
ETS	2022	May	5.32	-56.00	66.64
ETS	2022	Jun	5.57	-57.52	68.66
ETS	2022	Jul	7.90	-56.90	72.70
ETS	2022	Aug	9.41	-57.06	75.89
ETS	2022	Sep	10.44	-57.66	78.55
ETS	2022	Oct	20.84	-48.86	90.54
ETS	2022	Nov	29.84	-41.42	101.09
ETS	2022	Dec	18.29	-54.49	91.07
ETS	2023	Jan	10.39	-63.88	84.67
ETS	2023	Feb	7.91	-67.83	83.64
ETS	2023	Mar	7.17	-70.01	84.34
ETS	2023	Apr	5.49	-73.09	84.08
ETS	2023	May	5.32	-74.65	85.29
ETS	2023	Jun	5.57	-75.76	86.90
ETS	2023	Jul	7.90	-74.77	90.57
ETS	2023	Aug	9.41	-74.57	93.40
ETS	2023	Sep	10.44	-74.84	95.72
ETS	2023	Oct	20.84	-65.72	107.40
ETS	2023	Nov	29.84	-57.98	117.65
ETS	2023	Dec	18.29	-70.77	107.35
TBATS	2021	Jan	3.76	-9.70	17.22
TBATS	2021	Feb	8.97	-8.94	26.88
TBATS	2021	Mar	8.94	-10.06	27.94
TBATS	2021	Apr	8.01	-11.70	27.72
TBATS	2021	May	8.16	-12.18	28.49
TBATS	2021	Jun	8.34	-12.51	29.20
TBATS	2021	Jul	11.68	-9.67	33.02
TBATS	2021	Aug	12.44	-9.37	34.24
TBATS	2021	Sep	13.15	-9.06	35.35
TBATS	2021	Oct	24.80	2.21	47.39
TBATS	2021	Nov	32.35	9.38	55.32
TBATS	2021	Dec	22.11	-1.21	45.42
TBATS	2022	Jan	12.07	-11.55	35.70
TBATS	2022	Feb	10.77	-13.14	34.68
TBATS	2022	Mar	9.16	-15.04	33.36
TBATS	2022	Apr	8.18	-16.34	32.71
TBATS	2022	May	8.30	-16.56	33.15
TBATS	2022	Jun	8.46	-16.69	33.61
TBATS	2022	Jul	11.77	-13.68	37.22
TBATS	2022	Aug	12.51	-13.24	38.26
TBATS	2022	Sep	13.20	-12.83	39.23
TBATS	2022	Oct	24.85	-1.46	51.16
TBATS	2022	Nov	32.39	5.79	58.98
TBATS	2022	Dec	22.14	-4.73	49.00

TBATS	2023	Jan	12.10	-15.01	39.20
TBATS	2023	Feb	10.79	-16.56	38.13
TBATS	2023	Mar	9.17	-18.41	36.76
TBATS	2023	Apr	8.20	-19.66	36.05
TBATS	2023	May	8.30	-19.83	36.44
TBATS	2023	Jun	8.46	-19.92	36.85
TBATS	2023	Jul	11.78	-16.87	40.42
TBATS	2023	Aug	12.51	-16.40	41.43
TBATS	2023	Sep	13.21	-15.95	42.37
TBATS	2023	Oct	24.85	-4.55	54.25
TBATS	2023	Nov	32.39	2.73	62.05
TBATS	2023	Dec	22.14	-7.76	52.03
SARIM-NNAR	2021	Jan	8.21	-15.04	29.99
SARIM-NNAR	2021	Feb	10.39	-14.15	34.51
SARIM-NNAR	2021	Mar	12.09	-12.15	36.92
SARIM-NNAR	2021	Apr	10.01	-13.97	34.40
SARIM-NNAR	2021	May	9.52	-15.18	33.73
SARIM-NNAR	2021	Jun	11.53	-27.51	36.18
SARIM-NNAR	2021	Jul	18.71	-36.98	43.28
SARIM-NNAR	2021	Aug	20.33	-45.13	42.27
SARIM-NNAR	2021	Sep	25.54	-49.25	46.21
SARIM-NNAR	2021	Oct	37.80	-46.21	53.62
SARIM-NNAR	2021	Nov	48.77	-43.74	71.57
SARIM-NNAR	2021	Dec	27.45	-52.77	48.62
SARIM-NNAR	2022	Jan	17.71	-52.62	46.37
SARIM-NNAR	2022	Feb	19.41	-51.73	46.40
SARIM-NNAR	2022	Mar	21.13	-54.94	46.49
SARIM-NNAR	2022	Apr	19.49	-51.28	46.63
SARIM-NNAR	2022	May	18.87	-53.12	45.94
SARIM-NNAR	2022	Jun	20.24	-51.95	44.79
SARIM-NNAR	2022	Jul	25.70	-50.67	50.24
SARIM-NNAR	2022	Aug	27.21	-52.39	50.27
SARIM-NNAR	2022	Sep	29.76	-52.83	48.34
SARIM-NNAR	2022	Oct	39.19	-50.97	56.83
SARIM-NNAR	2022	Nov	48.31	-52.57	74.74
SARIM-NNAR	2022	Dec	32.53	-50.48	51.77
SARIM-NNAR	2023	Jan	23.89	-52.34	50.41
SARIM-NNAR	2023	Feb	24.73	-51.53	50.39
SARIM-NNAR	2023	Mar	25.13	-51.23	50.39
SARIM-NNAR	2023	Apr	23.81	-52.22	48.83
SARIM-NNAR	2023	May	23.79	-51.20	50.04
SARIM-NNAR	2023	Jun	25.68	-51.68	49.21
SARIM-NNAR	2023	Jul	29.25	-50.39	50.93
SARIM-NNAR	2023	Aug	28.78	-51.90	51.15
SARIM-NNAR	2023	Sep	30.85	-51.98	49.80
SARIM-NNAR	2023	Oct	39.79	-53.13	59.81
SARIM-NNAR	2023	Nov	48.79	-52.07	77.72
SARIM-NNAR	2023	Dec	35.75	-51.96	54.75
SARIMA-ETS	2021	Jan	4.73	-15.04	26.13

SARIMA-ETS	2021	Feb	5.15	-14.15	29.52
SARIMA-ETS	2021	Mar	6.18	-19.04	33.37
SARIMA-ETS	2021	Apr	4.58	-24.61	35.60
SARIMA-ETS	2021	May	5.08	-28.24	38.87
SARIMA-ETS	2021	Jun	7.22	-31.11	42.25
SARIMA-ETS	2021	Jul	11.37	-31.66	47.46
SARIMA-ETS	2021	Aug	11.68	-32.83	51.65
SARIMA-ETS	2021	Sep	14.16	-34.32	55.21
SARIMA-ETS	2021	Oct	28.33	-26.32	67.99
SARIMA-ETS	2021	Nov	41.80	-19.59	79.26
SARIMA-ETS	2021	Dec	24.55	-33.31	69.89
SARIMA-ETS	2022	Jan	9.23	-43.30	64.08
SARIMA-ETS	2022	Feb	8.17	-47.79	63.60
SARIMA-ETS	2022	Mar	8.31	-50.47	64.80
SARIMA-ETS	2022	Apr	6.16	-54.01	65.00
SARIMA-ETS	2022	May	6.33	-56.00	66.64
SARIMA-ETS	2022	Jun	8.26	-57.52	68.66
SARIMA-ETS	2022	Jul	12.29	-56.90	72.70
SARIMA-ETS	2022	Aug	12.53	-57.06	75.89
SARIMA-ETS	2022	Sep	14.97	-57.66	78.55
SARIMA-ETS	2022	Oct	29.10	-48.86	90.54
SARIMA-ETS	2022	Nov	42.56	-41.42	101.09
SARIMA-ETS	2022	Dec	25.30	-54.49	91.07
SARIMA-ETS	2023	Jan	9.97	-63.88	84.67
SARIMA-ETS	2023	Feb	8.91	-67.83	83.64
SARIMA-ETS	2023	Mar	9.05	-70.01	84.34
SARIMA-ETS	2023	Apr	6.90	-73.09	84.08
SARIMA-ETS	2023	May	7.07	-74.65	85.29
SARIMA-ETS	2023	Jun	9.00	-75.76	86.90
SARIMA-ETS	2023	Jul	13.02	-74.77	90.57
SARIMA-ETS	2023	Aug	13.26	-74.57	93.40
SARIMA-ETS	2023	Sep	15.70	-74.84	95.72
SARIMA-ETS	2023	Oct	29.83	-65.72	107.40
SARIMA-ETS	2023	Nov	43.29	-57.98	117.65
SARIMA-ETS	2023	Dec	26.03	-70.77	107.35
SARIMA-TBATS	2021	Jan	1.42	-15.04	17.22
SARIMA-TBATS	2021	Feb	5.68	-14.15	26.88
SARIMA-TBATS	2021	Mar	7.07	-12.15	27.94
SARIMA-TBATS	2021	Apr	5.84	-13.97	27.72
SARIMA-TBATS	2021	May	6.50	-12.89	28.49
SARIMA-TBATS	2021	Jun	8.60	-12.51	29.20
SARIMA-TBATS	2021	Jul	13.26	-9.67	33.02
SARIMA-TBATS	2021	Aug	13.19	-9.37	34.24
SARIMA-TBATS	2021	Sep	15.52	-9.06	35.69
SARIMA-TBATS	2021	Oct	30.31	2.21	53.62
SARIMA-TBATS	2021	Nov	43.06	9.38	71.57
SARIMA-TBATS	2021	Dec	26.46	-1.21	48.62
SARIMA-TBATS	2022	Jan	10.07	-11.55	35.70
SARIMA-TBATS	2022	Feb	9.60	-13.14	34.68

SARIMA-TBATS	2022	Mar	9.31	-15.04	33.36
SARIMA-TBATS	2022	Apr	7.51	-16.34	32.71
SARIMA-TBATS	2022	May	7.82	-16.56	33.15
SARIMA-TBATS	2022	Jun	9.71	-16.69	33.61
SARIMA-TBATS	2022	Jul	14.23	-13.68	37.22
SARIMA-TBATS	2022	Aug	14.08	-13.24	38.26
SARIMA-TBATS	2022	Sep	16.35	-12.83	39.23
SARIMA-TBATS	2022	Oct	31.11	-1.46	56.83
SARIMA-TBATS	2022	Nov	43.83	5.79	74.74
SARIMA-TBATS	2022	Dec	27.23	-4.73	51.77
SARIMA-TBATS	2023	Jan	10.82	-15.01	39.20
SARIMA-TBATS	2023	Feb	10.35	-16.56	38.13
SARIMA-TBATS	2023	Mar	10.05	-18.41	36.76
SARIMA-TBATS	2023	Apr	8.25	-19.66	36.05
SARIMA-TBATS	2023	May	8.56	-19.83	36.44
SARIMA-TBATS	2023	Jun	10.44	-19.92	36.85
SARIMA-TBATS	2023	Jul	14.96	-16.87	40.42
SARIMA-TBATS	2023	Aug	14.81	-16.40	41.43
SARIMA-TBATS	2023	Sep	17.08	-15.95	42.37
SARIMA-TBATS	2023	Oct	31.84	-4.55	59.81
SARIMA-TBATS	2023	Nov	44.57	2.73	77.72
SARIMA-TBATS	2023	Dec	27.96	-7.76	54.75
NNAR-ETS	2021	Jan	13.74	-5.35	31.01
NNAR-ETS	2021	Feb	12.76	-13.71	34.48
NNAR-ETS	2021	Mar	12.41	-19.04	35.55
NNAR-ETS	2021	Apr	10.54	-24.61	35.60
NNAR-ETS	2021	May	9.60	-28.24	38.87
NNAR-ETS	2021	Jun	9.87	-31.11	42.25
NNAR-ETS	2021	Jul	14.59	-31.66	47.46
NNAR-ETS	2021	Aug	17.11	-32.83	51.65
NNAR-ETS	2021	Sep	20.99	-34.32	55.21
NNAR-ETS	2021	Oct	30.14	-26.32	67.99
NNAR-ETS	2021	Nov	37.16	-19.59	79.26
NNAR-ETS	2021	Dec	21.10	-33.31	69.89
NNAR-ETS	2022	Jan	18.37	-43.30	64.08
NNAR-ETS	2022	Feb	18.29	-47.79	63.60
NNAR-ETS	2022	Mar	18.82	-50.47	64.80
NNAR-ETS	2022	Apr	17.86	-54.01	65.00
NNAR-ETS	2022	May	17.14	-56.00	66.64
NNAR-ETS	2022	Jun	16.93	-57.52	68.66
NNAR-ETS	2022	Jul	20.44	-56.90	72.70
NNAR-ETS	2022	Aug	23.60	-57.06	75.89
NNAR-ETS	2022	Sep	25.49	-57.66	78.55
NNAR-ETS	2022	Oct	30.93	-48.86	90.54
NNAR-ETS	2022	Nov	35.96	-41.42	101.09
NNAR-ETS	2022	Dec	25.14	-54.49	91.07
NNAR-ETS	2023	Jan	24.11	-63.88	84.67
NNAR-ETS	2023	Feb	23.79	-67.83	83.64
NNAR-ETS	2023	Mar	23.04	-70.01	84.34

NNAR-ETS	2023	Apr	22.51	-73.09	84.08
NNAR-ETS	2023	May	21.91	-74.65	85.29
NNAR-ETS	2023	Jun	22.36	-75.76	86.90
NNAR-ETS	2023	Jul	24.07	-74.77	90.57
NNAR-ETS	2023	Aug	25.10	-74.57	93.40
NNAR-ETS	2023	Sep	25.77	-74.84	95.72
NNAR-ETS	2023	Oct	30.88	-65.72	107.40
NNAR-ETS	2023	Nov	35.64	-57.98	117.65
NNAR-ETS	2023	Dec	27.67	-70.77	107.35
NNAR-TBATS	2021	Jan	10.34	-9.70	31.45
NNAR-TBATS	2021	Feb	13.24	-8.94	35.14
NNAR-TBATS	2021	Mar	13.18	-10.06	35.98
NNAR-TBATS	2021	Apr	11.06	-11.70	33.36
NNAR-TBATS	2021	May	10.06	-14.90	31.69
NNAR-TBATS	2021	Jun	10.13	-35.02	33.59
NNAR-TBATS	2021	Jul	16.53	-68.68	41.88
NNAR-TBATS	2021	Aug	19.38	-76.35	42.88
NNAR-TBATS	2021	Sep	23.04	-82.59	45.13
NNAR-TBATS	2021	Oct	31.50	-79.01	51.66
NNAR-TBATS	2021	Nov	36.20	-72.70	55.32
NNAR-TBATS	2021	Dec	27.00	-84.08	45.42
NNAR-TBATS	2022	Jan	21.53	-85.26	46.47
NNAR-TBATS	2022	Feb	20.91	-83.51	47.35
NNAR-TBATS	2022	Mar	20.08	-85.47	47.40
NNAR-TBATS	2022	Apr	18.40	-84.75	46.64
NNAR-TBATS	2022	May	17.27	-84.19	47.01
NNAR-TBATS	2022	Jun	16.73	-84.93	45.57
NNAR-TBATS	2022	Jul	21.49	-85.89	47.97
NNAR-TBATS	2022	Aug	24.34	-85.12	48.80
NNAR-TBATS	2022	Sep	25.86	-85.49	49.43
NNAR-TBATS	2022	Oct	32.00	-87.38	51.88
NNAR-TBATS	2022	Nov	35.84	-84.27	58.98
NNAR-TBATS	2022	Dec	30.21	-84.91	50.29
NNAR-TBATS	2023	Jan	25.19	-86.12	52.34
NNAR-TBATS	2023	Feb	24.54	-86.32	49.96
NNAR-TBATS	2023	Mar	23.73	-86.61	49.85
NNAR-TBATS	2023	Apr	23.01	-86.25	49.24
NNAR-TBATS	2023	May	22.79	-87.71	49.93
NNAR-TBATS	2023	Jun	22.68	-86.04	50.23
NNAR-TBATS	2023	Jul	25.05	-85.14	49.79
NNAR-TBATS	2023	Aug	25.74	-86.16	50.65
NNAR-TBATS	2023	Sep	26.18	-86.94	50.82
NNAR-TBATS	2023	Oct	32.03	-86.38	54.25
NNAR-TBATS	2023	Nov	35.80	-86.75	62.05
NNAR-TBATS	2023	Dec	30.63	-85.76	52.03
ETS-TBAT	2021	Jan	7.07	-9.70	26.13
ETS-TBAT	2021	Feb	8.44	-13.71	29.52
ETS-TBAT	2021	Mar	8.05	-19.04	33.37
ETS-TBAT	2021	Apr	6.75	-24.61	35.60

ETS-TBAT	2021	May	6.74	-28.24	38.87
ETS-TBAT	2021	Jun	6.96	-31.11	42.25
ETS-TBAT	2021	Jul	9.79	-31.66	47.46
ETS-TBAT	2021	Aug	10.92	-32.83	51.65
ETS-TBAT	2021	Sep	11.80	-34.32	55.21
ETS-TBAT	2021	Oct	22.82	-26.32	67.99
ETS-TBAT	2021	Nov	31.09	-19.59	79.26
ETS-TBAT	2021	Dec	20.20	-33.31	69.89
ETS-TBAT	2022	Jan	11.23	-43.30	64.08
ETS-TBAT	2022	Feb	9.34	-47.79	63.60
ETS-TBAT	2022	Mar	8.16	-50.47	64.80
ETS-TBAT	2022	Apr	6.84	-54.01	65.00
ETS-TBAT	2022	May	6.81	-56.00	66.64
ETS-TBAT	2022	Jun	7.01	-57.52	68.66
ETS-TBAT	2022	Jul	9.83	-56.90	72.70
ETS-TBAT	2022	Aug	10.96	-57.06	75.89
ETS-TBAT	2022	Sep	11.82	-57.66	78.55
ETS-TBAT	2022	Oct	22.84	-48.86	90.54
ETS-TBAT	2022	Nov	31.11	-41.42	101.09
ETS-TBAT	2022	Dec	20.21	-54.49	91.07
ETS-TBAT	2023	Jan	11.24	-63.88	84.67
ETS-TBAT	2023	Feb	9.35	-67.83	83.64
ETS-TBAT	2023	Mar	8.17	-70.01	84.34
ETS-TBAT	2023	Apr	6.84	-73.09	84.08
ETS-TBAT	2023	May	6.81	-74.65	85.29
ETS-TBAT	2023	Jun	7.02	-75.76	86.90
ETS-TBAT	2023	Jul	9.84	-74.77	90.57
ETS-TBAT	2023	Aug	10.96	-74.57	93.40
ETS-TBAT	2023	Sep	11.83	-74.84	95.72
ETS-TBAT	2023	Oct	22.84	-65.72	107.40
ETS-TBAT	2023	Nov	31.11	-57.98	117.65
ETS-TBAT	2023	Dec	20.21	-70.77	107.35
